# NK-/T-cell lymphomas

**DOI:** 10.1038/s41375-021-01313-2

**Published:** 2021-06-11

**Authors:** Hua Wang, Bi-bo Fu, Robert Peter Gale, Yang Liang

**Affiliations:** 1 Department of Hematologic Oncology, State Key Laboratory of Oncology in South China, Collaborative Innovation Center for Cancer Medicine, Sun Yat-sen University Cancer Center, Guangzhou, PR China; 2 Haematology Research Centre, Department of Immunology and Inflammation, Imperial College London, London, UK

**Keywords:** Prognosis, Cancer

## Abstract

Natural killer/T-cell lymphoma (NKTL) is a sub-type of Epstein–Barr virus (EBV)-related non-Hodgkin lymphomas common in Asia and Latin America but rare elsewhere. Its pathogenesis is complex and incompletely understood. Lymphoma cells are transformed from NK- or T-cells, sometimes both. EBV-infection and subsequent genetic alterations in infected cells are central to NKTL development. Hemophagocytic syndrome is a common complication. Accurate staging is important to predict outcomes but there is controversy which system is best. More than two-thirds of NKTL lympohmas are localized at diagnosis, are frequently treated with radiation therapy only and have 5-year survival of about 70 percent. Persons with advanced NKTLs receive radiation therapy synchronously or metachronously with diverse multi-drug chemotherapy typically including l-asparginase with 5-year survival of about 40 percent. Some persons with widespread NKTL receive chemotherapy only. There are few data on safety and efficacy of high-dose therapy and a haematopoietic cell autotransplant. Immune therapies, histone deacetylase (HDAC)-inhibitors and other drugs are in early clinical trials. There are few randomized controlled clinical trials in NKTLs and no therapy strategy is clearly *best*; more effective therapy(ies) are needed. Some consensus recommendations are not convincingly evidence-based. Mechanisms of multi-drug resistance are considered. We discuss these issues including recent advances in our understanding of and therapy of NKTLs.

## Introduction

Natural killer/T-cell lymphoma (NKTL), an Epstein–Barr virus (EBV) associated lympho-proliferative disease, accounts for <2 percent of T-cell lymphomas. It develops from transformation of natural killer (NK)-cells or cytotoxic T-cells, rarely both. NKTL is relatively common in Asia and, to lesser extent, in Latin America, but rare in Europe and North America. NKTLs can be nodal (nNKTL) or extra-nodal (eNKTL), forms which differ substantially in clinical, patho-physiological and genetic features [1–3].

In 2016 WHO scheme NKTLs are classified as EBV-associated T- and NK-cell lympho-proliferative diseases [4]. Most eNKTLs occur in the nasal cavity and are termed nasal-type eNKTL but can occur at any site. Localized NKTL is curable but most persons with advanced disease have a poor prognosis [5–7].

Diagnosis of NKTL depends on histology including T- and/or NK-cells expressing CD3ε, granzyme B, perforin, TIA1, and EBV [8]. Cytogenetic abnormalities include del(6), del(8) and del(14). Commonly mutated genes include *TP53, DDX3X, STAT3, JAK3, MGA, BCOR, ECSIT*, and *MCL1* [9, 10].

Several prognostic and predictive models of NKTLs are proposed including International Prognostic Index (IPI), Korean Prognostic Index (KPI), Prognostic Index of Natural Killer Lymphoma (PINK), and Nomogram-revised Risk Index (NRI). None is sufficiently accurate to predict outcomes at the patient level [11–14].

Localized NKTL is often treated with radiation therapy with 5-year survival of about 70% [5]. Advanced and recurrent NKTLs are typically treated with radiation therapy and multi-drug chemotherapy regimens including l-asparaginase with 5-year survival only about 40% [7]. High-dose therapy and autologous hematopoietic cell transplants are sometimes used but mostly ineffective and better therapies are needed [15, 16]. Immune therapies directed against NKTL-associated targets such as immune checkpoint inhibitors, anti-CD30 and -CD38 monoclonal antibodies and against EBV-related targets are increasingly used [17–20]. We use a question-and-answer format to summarize major issues in the biology and therapy of NKTLs.

## Why the geographic diversity of NKTLs?

NKTL is relatively common in Asia and Latin America compared with Europe and North America [21–23]. Why is incompletely understood but important considerations include EBV type and genetic susceptibility, probably both [24–28].

EBV is divided into two major strains or clades, EBV strain or type -1 and EBV strain or type-2 which have different EBNA-2 and -3A, 3B, and 3C genes with different transforming and reactivation activities [29, 30]. EBV type-1 is dominant globally [30–33]. EBV starin-1 is common in NKTLs compared with EBV strain-2 [25, 34]. These data mean geographic differences in EBV type prevalences only partly explain differences in geographic incidences of NKTLs. Several other EBV strains and inter-starin viruses have been isolated from human cancers, especially nasopharyngeal cancinomas, and lymphomas including NKTLs. EBV strains associated with nasopharyngeal cancinomas include GD1, GD2, HKNPC1, C666-1, and M81 [29, 35–40]. whereas EBV viruses associated with Burkitt lymphoma include AG876, Akata, and Mutue [29, 41–44].

Many EBV strains identified in different geographic areas differ mainly in *LMP1* genes [29, 34]. The Asian EBV strains were isolated from samples in East and South Asia and clearly differ from strains from other parts of the world [29]. Recent phylogenetic analyses indicate EBV genomes sequenced from Asians with NKTL are closer to the Asian than non-Asian strains indicating an association between Asian strains and NKTL and a potential explanation of the geographic diversity of NKTLs [29, 45].

Genomic analyses indicate several variants of EBV derived from NKTLs [38, 46, 47]. The frequent single nucleotide variant was located at *BPLE1* gene [47]. A 30 base pair deletion in EBV *LMP1* gene C terminus region was common and associated with oncogenicity of EBV strain-2 in NKTLs [30, 46, 48]. Transcriptome analyses indicate differential expression of latent and lytic genes and variations of T-cell epitopes in EBV strains from NKTLs suggesting some specific EBV variants are more likely to cause NKTL [47].

Studies on race-related genetic predisposition to NKTL have been done in east Asia [28, 49]. Bei et al. reported correlations between the *HLADPB1* rs9277378 single nucleotide polymorphism locus and immune response to EBV-infection and likelihood of developing NKTL [49]. These authors also reported different *IL18RAP* rs13015714 genotypes promote cell proliferation and are associated with developing NKTL [28]. Another association with developing NKTL is rs9271588 at GRCh38 38.1/142 [28]. More studies of genetic predisposition to NKTL are needed.

## Are NK/T-cell lymphomas T- or NK-cell cancers or both?

NK- and T-cells derive from the same lymphoid progenitor cell which develops into different lineages because of expression of different transcription factors [50]. Most NKTLs originate in NK-cells with germline T-cell receptor (TCR) genes [1]. Rarely, NKTLs arise in T-cells with rearranged TCR genes. A study of 67 subjects with NKTL reported more than two-thirds of cases were of NK-cell origin [51]. NK-cells express perforin, granzyme B, TIA1, CD2, cytoplasmic CD3ε, and CD56, expressed in most extra-nodal NKTLs [8, 52]. Granzyme B, TIA1, CD2, CD56 are also sometimes expressed in T-cell-derived cases. However, TCR protein is only expressed in T-cell cases [51, 53]. Del(14q11.2) is a potential indicator of a T-cell origin of nodal NKTLs [2, 54]. NKTLs of NK-cell origin often have *STAT3*, *DDX3X* mutations whereas those of T-cell origin often have *TP53* and *EPHA1* mutations [9]. Clinical features and therapy response of NK- and T-cell origin NKTLs are similar [4, 51, 53, 54]. Nodal NKTLs are included into EBV-positive variant of peripheral T-cell lymphoma not otherwise specified (NOS) [3].

## What is the most accurate NKTL staging system?

The Ann Arbor staging system is the most widely used for staging NKTL. Although useful for planning radiation therapy it fails to consider the prognostic impact of certain anatomical sites such as the aero-digestive tract, local invasion, and regional lymph node involvement [55].

The TNM staging system is based on a single-center study of anthracycline chemotherapy in persons with nasal NKTL. Although it emphasizes the prognostic impact of regional lymph node involvement it is not intended for extra-nasal NKTLs and is not validated in persons receiving l-asparaginase therapy [56].

The Chinese Southwest Oncology Group and Asia Lymphoma Study Group ENKTL (CA) staging system considers site, local invasion, regional lymph node involvement and metastasis and is validated in persons receiving l-asparaginase [13]. This system seems more accurate than others in estimating survival and operates under different therapies. Table 1 summarizes the details of the CA system. Other co-variates which may improve the accuracy of staging systems include measures of circulating EBV-DNA and positron emission tomography/computed tomography scanning [5, 57–59].

**Table 1:** Detailed description of The Chinese Southwest Oncology Group and Asia Lymphoma Study Group (CA) ENKTL staging system.

Stage	Description
I	Lesions confined to the nasal cavity or nasopharynx
	No local invasion
	No lymph node involvement
II	Lesions confined to the nasal cavity or nasopharynx
	Local invasion
	No lymph node involvement
	Non-nasal disease
III	Lesions with regional lymph node involvement
IV	Non-regional lymph node involvement
	Lymph nodes above and below diaphragm
	Widespread disease

## Which is the best survival prognostic/predictive model?

The IPI, KPI, and the PINK are the most widely used NKTL prognostic and/or predictive models. Accuracy of the IPI was confirmed in many studies of low-and high-grade lymphomas including studies which included l-asparaginase therapy but accuracy in NKTL is controversial [11]. Although most persons with stage-I/-II NKTL are classified as low-risk in the IPI some have a poor prognosis [12, 60].

Accuracy of the KPI model is validated in persons receiving l-asparginase. However, there is no preditive discrimaination between persons in the IPI low- and low-intermediate risk cohorts or between persons in the IPI high-intermediate and high-risk cohorts [60]. A multi-variable Cox regression analyses which included local invasion and the KPI reported local invasion was a better survival predictor than the KPI only [12]. An important limitation is the IPI and KPI models were developed before l-asparginase was widely used [60, 61]. In contrast, the PINK model was developed and validated in persons receiving l-asparginase-based therapy and more accurately identifies prognoses of persons in the same risk cohorts in the IPI and KPI models but is also far from accurate [13, 62].

Li et al. used Ann Arbor stage, age, Eastern Cooperative Oncology Group performance score, lactate dehydrogenase (LDH) and local invasion to develop a predictive nomogram and NRI for NKTL [14, 63]. The NRI is more accurate compared with the IPI, KPI, or PINK models [14]. These models are compared in Table 2.

**Table 2:** Comparison of prognostic/predictive models.

Model	Prognostic factors (points)	Definition (points)	Development/validation era		Accuracy^a^	
			Anthracycline	No anthracycline	C-statistic	AUC for 5-year survival
IPI	Age >60 years (1); Increased LDH (1); PS ≥ 2 (1); Stage-III/IV (1); Extra-nodal disease ≥2 (1)	Low-risk (0–1) Intermediate low-risk (2) Intermediate high-risk (3) High-risk (≥4)	Development	–	0.62	0.61
KPI	B-symptoms (1); Stage-III/IV (1); Increased LDH (1); Regional lymph node involvement (1)	Group 1 (0) Group 2 (1) Group 3 (2) Group 4 (≥3)	Development	Validation	0.64	0.68
PINK	Age >60 years (1); Stage-III/IV (1); Distant lymph node involvement (1); Non-nasal disease (1)	Low-risk (0) Intermediate-risk (1) High-risk (≥2)	–	Development and validation	0.61	0.63
NRI	Age >60 years (1); Stage-II (1); Stage-III/IV (2); PS ≥ 2 (1); Increased LDH (1); With PTI (1)	Low-risk (0) Intermediate low-risk (1) Intermediate high-risk (2) High-risk (3) Very high-risk (≥4)	Development	Validation	0.70	0.72

*AUC* area under curve, *LDH* lactate dehydrogenase, *PS* performance status, *PTI* primary tumor invasion.

^a^Ref. [14].

## What is the best therapy of early-stage NKTL?

Data from 2 recent retrospective studies indicate radiation therapy alone is effective therapy of early-stage NKTL with no benefit of adding chemotherapy. In these studies outcomes with different therapies were compared using a propensity score-matched analysis [6, 64]. Subjects in the low-risk cohort had favorable outcomes with radiation therapy alone with 5-year survival of about 90 percent [6, 64, 65]. Similar data are reported for subjects staged using the CA system [13]. Others suggest risk-adapted therapy for early-stage NKTL where chemotherapy is given only persons with an adverse prognosis despite being low-risk [5,65–67].

## What is the best therapy of advanced NKTL?

There are consensus persons with advanced NKTL benefit from combined radiation therapy and chemotherapy. For example, in a study using the NRI subjects in the intermediate-/high-risk cohorts benefited from combined radiation therapy and chemotherapy compared with radiation therapy only with 5-year survivals of about 75% versus 60% [6, 65, 68].

Similar data are reported for subjects staged using the CA system [13]. The conclusion adding chemotherapy to radiation benefits intermediate/high stage subjects are not from a randomized controlled trial and should be viewed cautiously.

Combined therapy can be given synchronously or given metachronously in either order [5]. In the synchronous format radiation therapy is given with reduced doses of DeVIC (dexamethasone, etoposide, ifosfamide, and carboplatin), ESHAP (etoposide, dexamethasone, high-dose cytarabine, and cisplatin), or DEP (dexamethasone, etoposide, and cisplatin) [69–72]. Also, radiation therapy can be given with weekly cisplatin followed by VIPD (etoposide, ifosfamide, cisplatin, and dexamethasone) or VIDL (etoposide, ifosfamide, dexamethasone, and l-asparaginase) [73, 74]. Data from metachronous format include DDGP (pegaspargase, gemcitabine, cisplatin, and dexamethasone) followed by radiation therapy with little toxicity [75]. SMILE (dexamethasone, methotrexate, ifosfamide, l-asparaginase, and etoposide) followed by radiation therapy is also effective [7]. Another metachronous format is to sandwich radiation between chemotherapy courses. Examples include MESA (methotrexate, etoposide, dexamethasone, and pegaspargase) followed by radiation and radiation therapy combined with GELOX (gemcitabine, l-asparaginase and oxaliplatin) or PGEMOX (gemcitabine, pegaspargase, and oxaliplatin) [76–78].

Persons with widespread NKTL are sometimes receive chemotherapy only. Most trials had few, selected subjects with wide confidence intervals precluding critical interpretation or comparison. Regimens tested included SMILE, PEMD (pegylated-L-asparginase, etoposide, methotrexate, and dexamethasone), AspaMetDex (l-asparaginase, methotrexate, and dexamethasone), PGEMOX, and GDP (gemcitabine, dexamethasone, and cisplatin) [79–83]. A randomized controlled trial compared outcomes of DDGP and SMILE. DDGP had better survival with less severe adverse events [84]. These regimens are displayed in Table 3. SMILE, PGEMOX, and DDGP are recommended in the NCCN guidelines with AspaMetDex is recommended for persons unable to tolerate intensive therapy [16].

**Table 3:** Therapies for advanced NKTL.

Regimen	*N* subjects	Response	Survival (95%CI)	Adverse events (Grades-3/-4)				Ref.
				↓ WBC	Anemia	↓ Platelets	Liver	
CCRT-DeVIC	27	20 CR 1 PR	2-year 78% (57, 89%) 5-year 70% (49, 84%)	100%	15%	11%	NA	[69, 70]
CCRT-ESHAP	13	12 CR 1 PR	2-year 72% (35, 90%)	92%	77%	69%	0	[71]
CCRT-DEP ± DVIP	33	20 CR 6 PR	5-year 66% (50, 83%)	83%	28%	21%	9%	[72]
CCRT-VIPD	30	24 CR 1 PR	3-year 86% (NA)	47%	27%	23%	NA	[73]
CCRT-VIDL	30	26 CR 1 PR	5-year 73% (NA)	80%	10%	13%	10%	[74]
SCRT-DDGP	30	22 CR 3 PR	5-year 86% (NA)	20%	20%	27%	3%	[75]
SCRT/Sandwich-SMILE	17	14 CR	NA	NA	NA	NA	NA	[7]
Sandwich-MESA	40	34 CR 1 PR	2-year 92% (NA)	53%	13%	0	NA	[76]
Sandwich-GELOX/PGEMOX	27	20 CR 6 PR	5-year 85% (NA)	33%	7%	30%	4% ↑ transaminases 7% ↑ Bilirubin	[77, 78]
SMILE	38	17 CR 13 PR	1-year 55% (38, 69%)	100%	50%	64%	32% ↑ AST 32% ↑ ALT 11% ↑ Bilirubin	[79]
PEMD	32	15 CR 9 PR	4-year 51% (32, 70%)	34%	9%	9%	9%	[80]
AspaMetDex	19	11 CR 3 PR	NA^a^	42%^b^	21%	5%	16%	[81]
PGEMOX ± RT/Autotrasplant	35	18 CR 10 PR	3-year 65% (NA)	40%	26%	31%	12% ↑ transaminases 6% ↑ Bilirubin	[82]
GDP	41	17 CR 17PR	2-year 55% (NA)	34%^b^	15%	20%	0	[83]
DDGP	21	15 CR 5 PR	2-year 74% (NA)	62%	52%	62%	5%	[84]

*CI* 95% confidence interval, *CCRT* concurrent chemotherapy and radiation therapy, *SCRT* sequential chemotherapy and radiation therapy, *CR* complete remission, *PR* partial remission, *Pe* pegaspargase, *NA* not applicable, *AST* aspartate transaminase, *ALT* alanine transaminase.

^a^Median survival time of 12 months.

^b^Neutropenia.

Most data we cite for therapy of advanced NKTL are from retrospective or uncontrolled studies and report similar outcomes with synchronous and metachronous therapy and with diverse radiation therapy and chemotherapy regimens, formats, schedules etc [54, 85, 86]. There are also a myriad of radiation therapy doses, fields, and schedules. The many formats for radiation therapy and diverse chemotherapy regimens suggest no convincing data any strategy is *best*. Curiously, the NCCN guidelines recommend radiation therapy combined with DeVIC, VIPD, modified SMILE, or PGEMOX [16]. We think this recommendation is not evidence-based and should be viewed cautiously.

## Is there a role for high-dose therapy and a haematopoietic cell autotransplant in NKTL?

There are several small studies of high-dose chemotherapy followed by a haematopoietic cell autotransplant in persons with advanced NKTL. The largest included 31 subjects receiving an autotransplant as initial therapy. 3-year progression-free survival (PFS) and survival were 40% (95% Confidence Interval [CI], 22, 58%) and 52% (34, 71%) [87]. The largest retrospective study in subjects from Western countries reported comparable survival to Asian subjects with 2-year PFS of 33% (13, 84%) and survival of 40% (19, 86%) [15]. These data are largely uninterpretable because of huge confidence intervals. A multi-center phase-2 study evaluated VIDL followed by an autotrasplant in 27 subjects with advance NKTL. 8 of 17 responders after induction chemotherapy maintained a complete remission posttransplant but there were no controls [88]. These limited data from uncontrolled clinical trials provide no basis for recommending or not recommending autotransplants in advanced NKTL and are displayed in Table 4. Surprisingly, NCCN guidelines recommend haematopoietic cell_transplants for persons with advanced NKTL in complete remission or a biopsy-negative partial remission after first-line therapies. This recommendation seems based on few, data and is unconvincing.

**Table 4:** Haematopoietic cell autotransplants in NKTL.

*N* subjects	Pre-transplant Response	Post-transplant Response	PFS (95% CI)	OS (95% CI)	Ref.
31	16 CR 15 PR	19 CR 4 PR 6 PD 2 NA	3-year 40% (22, 58%)	3-year 52% (34, 71%)	[87]
10	6 CR 2 PR 2 PD	NA	2-year 33% (13, 84%)	2-year 40% (19, 86%)	[15]
17	17 CR/PR	8 CR 9 PD	NA	NA	[88]

*PFS* progression-free survival, *OS* overall survival, *CI* confidence interval, *CR* complete remission, *PR* partial remission, *PD* progressive disease, *NA* not applicable.

## What is the mechanism of multi-drug resistance (MDR) in NKTL?

Most NKTLs express *MDR1* which encodes the P-glycoprotein which mediates cell efflux of doxorubicin and vincristine from cells and may explain resistance of NKTLs to anthracycline-based therapy [89]. Response rates of *MDR1*-negative subjects to CHOP (cyclophosphamide, doxorubicin, vincristine, and prednisone) is significantly higher than those *MDR1*-positive in 1 study but not others [90]. Interleukin-13 (IL-13) and *ABCC4* are highly expressed in NKTLs and also contributes to doxorubicin resistance [91]. Wang et al. reported a higher concentration of IL-2 receptors α (IL-2R α) on NKTL cell lines compared with normal NK-cells [92]. Over-expression of IL-2R α is associated with resistance to killing by gemcitabine, doxorubicin, and l-asparaginase. *MY*C expression promotes expression of SNHG12, the small nucleolar RNA host gene 12 of long non-coding RNAs which mediates resistance of NKTL cells to cisplatin [93]. Clinical translation of these data are lacking.

## Why is NKTL prone to hemophagocytic syndrome (HPS)?

HPS is an immune-mediated syndrome characterized by systemic activation of macrophages resulting in a hyper-inflammatory response and widespread tissue and organ damage. HPS is classified as primary or secondary. People with haematopoietic cancers, especially NKTLs, are at risk to develop secondary HPS. NKTL and HPS are associated with EBV infection [94]. In persons with EBV-associated NKTL TNF-α expression is increased activating monocyte/macrophage phagocytosis and cytokine secretion. *ECSIT*^T419C^ is common in NKTL [10]. A mutation in *ECSIT, ECSIT*^V140A^ combines with the S100A8/S100A9 hetero-dimer which promotes stable binding of arachidonic acid to S100A9 and contributes to the assembly of NADPH oxidase followed by activation of NF-κB pathway. These steps promote secretion of TNF-α and IFN-γ which activate macrophages and precipitate cytokine storm resulting in secondary HPS. Also, persons with advanced NKTL have high interleukin-18 (IL-18) concentrations which also correlate with high TNF-α and IFN-γ concentrations and are associated with lymphoma-associated hemophagocytic syndrome (LAHPS) [95]. One study reported bone marrow infiltration, hepato-splenomegaly, and high LDH levels were risk factors for LAHPS. The incidence of LAHPS in people with two or three risk factors was >60 percent [96].

## Is there a role for immune therapy of NKTL?

PD-L1 is expressed frequently on NKTL cells and is associated with poor prognosis making anti-PD-L1/PD-1 antibodies an attractive therapy [19, 97]. Pembrolizumab is reported effective in persons with advanced NKTL and there are encouraging results with nivolumab [98–101]. Other active drugs include sintilimab, CS-001, and avelumab [102–105]. These data are summarized in Table 5. Although pembrolizumab and nivolumab are recommended in the NCCN guidelines for advanced NKTL the strength of evidence is weak and they should probably only be used in the context of a clinical trial [16].

**Table 5:** Checkpoint-inhibitors in NKTL.

*N* Subjects		Response	Ref.
7	Pembrolizumab	5 CR/2 PR	[98]
7		2 CR/2 PR	[99]
14		5 CR/1 PR	[100]
3	Nivolumab	1 CR	[101]
28	Sintilimab	4 CR/15 PR	[103]
37	Sintilimab/Chidamide	16 CR/5 PR	[116]
6	Sintilimab/Chemotherapy	2 CR/4 PR	[102]
29	CS-001	7 CR/2 PR	[104]
21	Avelumab	5 CR/3 PR	[105]

*CR* complete remission, *PR* partial remission.

CD38 and CD30 are expressed in about one-half of NKTLs [106, 107]. There is a case report of efficacy of daratumumab, an anti-CD38 antibody, in advanced NKTL [108]. Preliminary safety and efficacy data are also reported from a multi-center phase-2 study (NCT02927925) [18]. Another study claimed efficacy of brentuximab vedotin, an anti-CD30 antibody, in 2 subjects [109, 110]. A phase-2 study of 7 subjects reported responses [17]. These data without controls are insufficient to comment critically on safety and efficacy of this approach and it should be used only in the context of clinical trials.

EBV-infection is associated with NKTLs and provides a therapy target. In one study 10 subjects in complete remission after diverse therapies received autologous cytotoxic T-cells against EBV latent membrane protein (LMP)-1/-2a with encouraging results. However, controls were lacking [20]. Kim et al. reported responses in four subjects with advanced NKTL receiving autologous EBV-specific T-cells [111]. Transfusing allogeneic EBV-specific T-cells from donors was reported to improve survival in persons at high risk of relapse compared with historical cohorts [112]. These few data and absent controls preclude critical analyses of safety and efficacy and suggest use only in the context of a clinical trial.

## Are histone deacetylase (HDAC)-inhibitors active in NKTL?

Chidamide, a selective inhibitor of HDAC1, 2, 3, and 10 was tested in a phase-2 study in 16 subjects with advanced NKTL. Three responded with one complete remission [113]. In another study subjects with advanced NKTL received chidamide only (*N* = 19) or with chemotherapy (*N* = 18). Responses were reported in five subjects receiving chidamide only and eight receiving combined therapy [114]. A phase-2 study reported data of 15 subjects with advanced NKTL receiving chidamide with 6 responders [115]. There were 21 responders among 37 subjects with advanced NKTL receiving chidamide in a phase 1b/−2 study [116]. Another study reported a response in a subject with advanced NKTL receiving panobinostat with bortezomib [117]. Other HDAC-inhibitors such as romidepsin and belinostat were used in phase-2 trials with too few subjects to comment critically [118–122]. Other data suggest vorinostat and valproic acid are active against NKTL cells in vitro and in animal models [123, 124]. Studies of HDAC-inhibitors are displayed in Table 6. These few data and absent controls preclude critical analyses of safety and efficacy and suggest use only in the context of a clinical trial.

**Table 6:** HDAC-inhibitors in NKTL.

*N* Subjects		Response	Ref.
16	Chidamide	1 CR/2 PR	[113]
19		2 CR/3 PR	[114]
18	Chidamide/Chemotherapy	2 CR/6 PR	
15	Chidamide	4 CR/2 PR	[115]
37	Chitamide/Sintilimab	16 CR/5 PR	[116]
2	Panobinostat/Bortezomib	1 PR	[117]
3	Romidepsin ± Chemotherapy	NA	[119, 121, 122]
2	Belinostat	1 CR/1 PR	[120]
1	Vorinostat	CR	[123]
1			[124]

*CR* complete remission, *PR* partial remission.

## Conclusion

There is substantial recent progress in understanding the biology of NKTLs combined with modest therapy advances. Staging and predictive and prognostic scoring models have improved but greater accuracy and precision are needed. Risk-adapted therapy is proposed but unvalidated. Radiation therapy has proved effective in localized NKTL whereas l-asparaginase based chemotherapy is effective in persons with advanced disease. No therapy format is proved best and randomized controlled trials are lacking. New therapy strategies including immune and targeted therapies are advancing in clinical trials. Progress, but more is needed.
